# Supplementary figures and images for: Gender differences in Leptospira exposure risk, perceptions of disease severity, and high-risk behaviours in Salvador, Brazil: A cross-sectional study
Source: PLOS Glob Public Health. 2025 Jun 27;5(6):e0004786. doi: 10.1371/journal.pgph.0004786 (PMC12204547; doi:10.1371/journal.pgph.0004786)

**S1 Checklist: PLOS Inclusivity in Global Research Checklist**


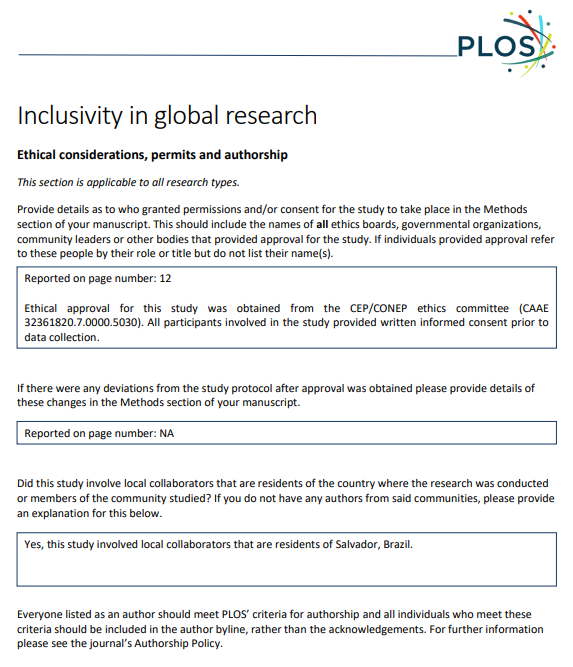


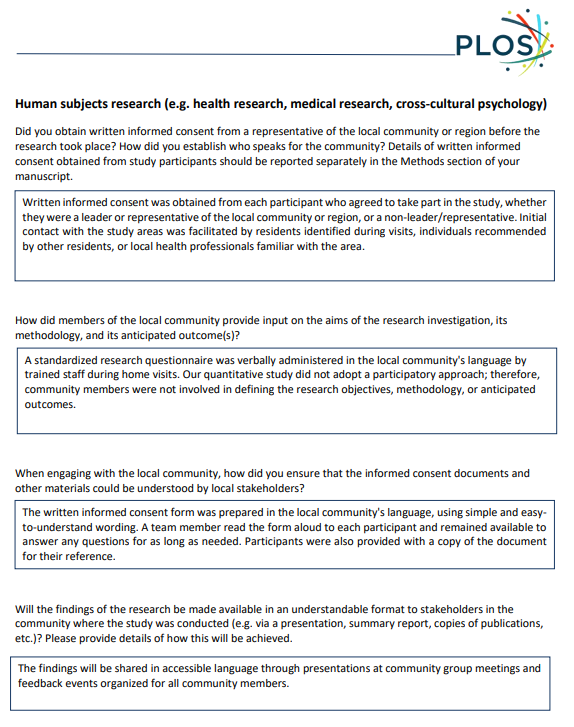


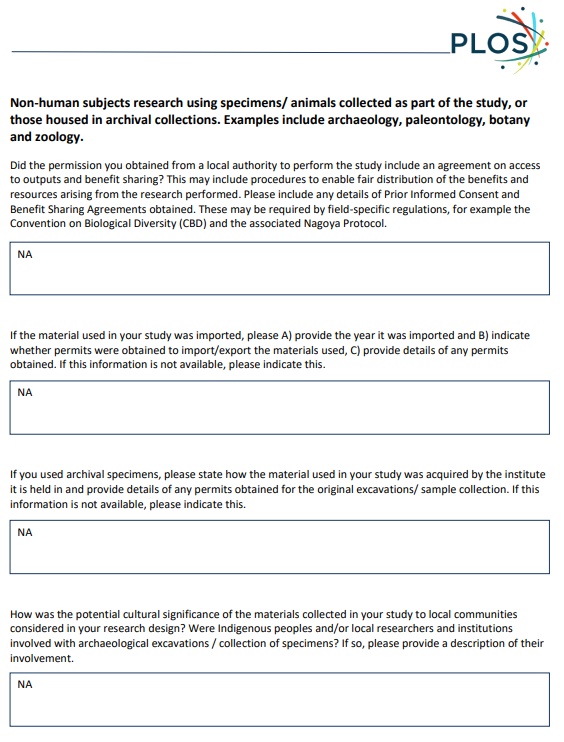


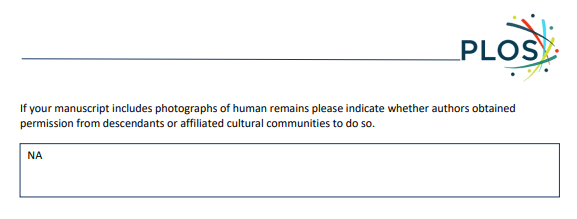

Supplement: S1 Checklist — (DOCX) [file pgph.0004786.s001.docx]
