# Supplementary material for: Gender differences in Leptospira exposure risk, perceptions of disease severity, and high-risk behaviours in Salvador, Brazil: A cross-sectional study
Source: PLOS Glob Public Health. 2025 Jun 27;5(6):e0004786. doi: 10.1371/journal.pgph.0004786 (PMC12204547; doi:10.1371/journal.pgph.0004786)
Supplement: S1 Fig — DWA: Domestic waste accumulation. < 10m from DWA and open sewer refer to household distance from risk factors. (DOCX) [file pgph.0004786.s002.docx]

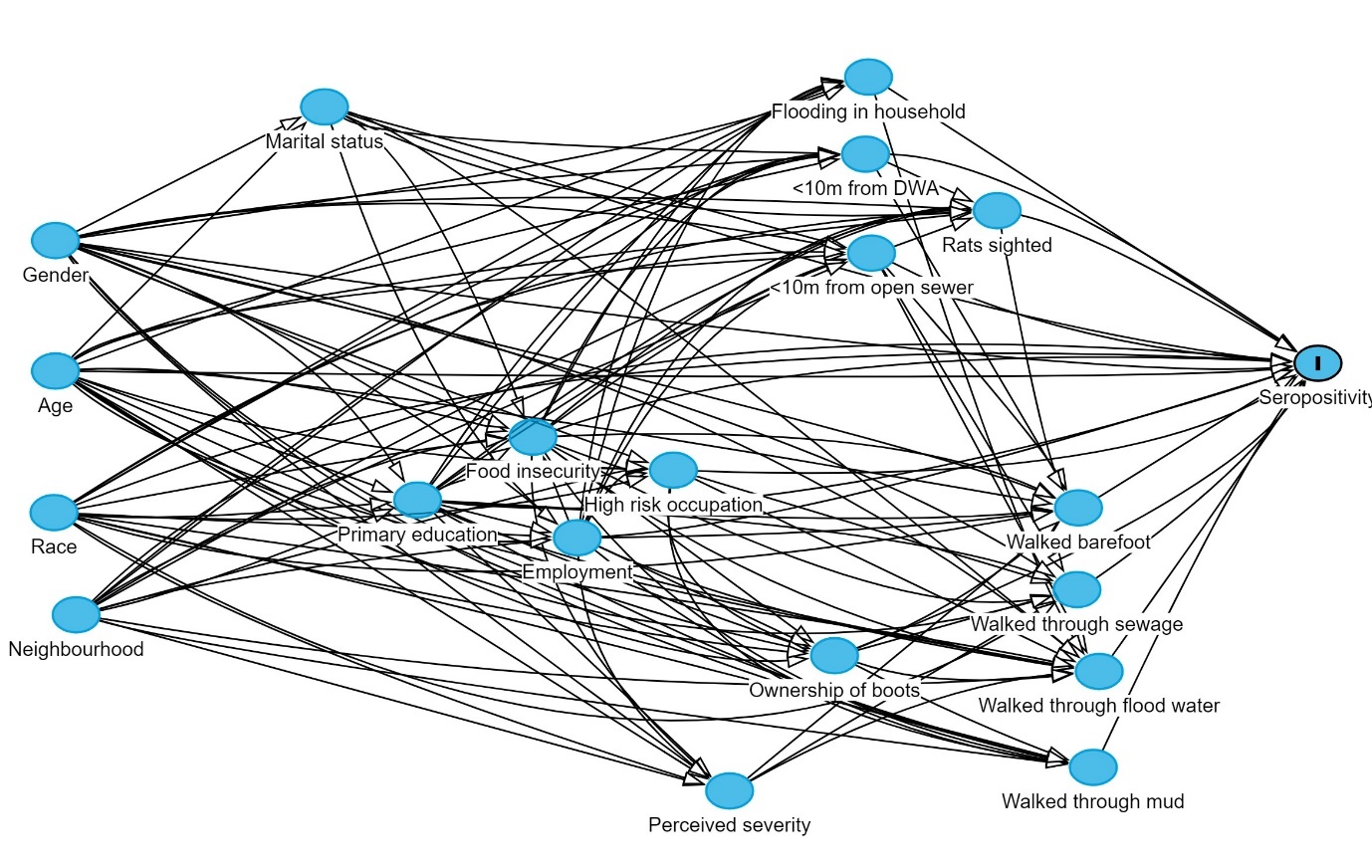
S1 Fig: Full version of the DAG used in analysis, also available at [https://dagitty.net/dags.html?id=XxPTXytr#](https://dagitty.net/dags.html?id=XxPTXytr). DWA: Domestic waste accumulation. <10m from DWA and open sewer refer to household distance from risk factors.
