# Supplementary material for: Gender differences in Leptospira exposure risk, perceptions of disease severity, and high-risk behaviours in Salvador, Brazil: A cross-sectional study
Source: PLOS Glob Public Health. 2025 Jun 27;5(6):e0004786. doi: 10.1371/journal.pgph.0004786 (PMC12204547; doi:10.1371/journal.pgph.0004786)

S2 Fig: GAMs of age with seroprevalence response variable. GAMs were built using univariable models. Shaded area corresponds to 95%CI.


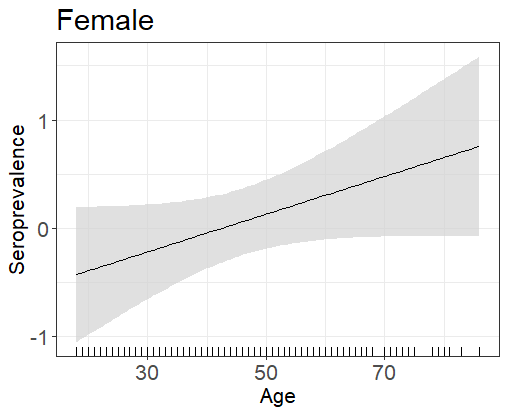

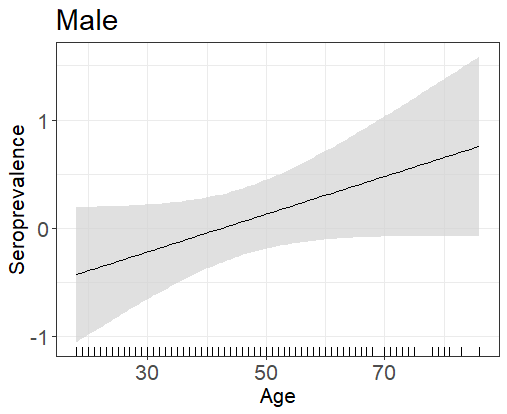

Supplement: S2 Fig — GAMs were built using univariable models. Shaded area corresponds to 95% CI. (DOCX) [file pgph.0004786.s003.docx]
