# Supplementary material for: Gender differences in Leptospira exposure risk, perceptions of disease severity, and high-risk behaviours in Salvador, Brazil: A cross-sectional study
Source: PLOS Glob Public Health. 2025 Jun 27;5(6):e0004786. doi: 10.1371/journal.pgph.0004786 (PMC12204547; doi:10.1371/journal.pgph.0004786)
Supplement: S4 Fig — Odds ratios are presented for females and males who perceived leptospirosis as extremely serious, compared to those of the same gender and age group who perceived leptospirosis as less serious. The plots are displayed on a log scale due to the high uncertainty in some of the estimates. (DOCX) [file pgph.0004786.s005.docx]

S4 Fig: Total causal effects of perceived severity with A) walking barefoot outside the home and B) walking through sewage water, stratified by age. Odds ratios are presented for females and males who perceived leptospirosis as extremely serious, compared to those of the same gender and age group who perceived leptospirosis as less serious. The plots are displayed on a log scale due to the high uncertainty in some of the estimates.

**
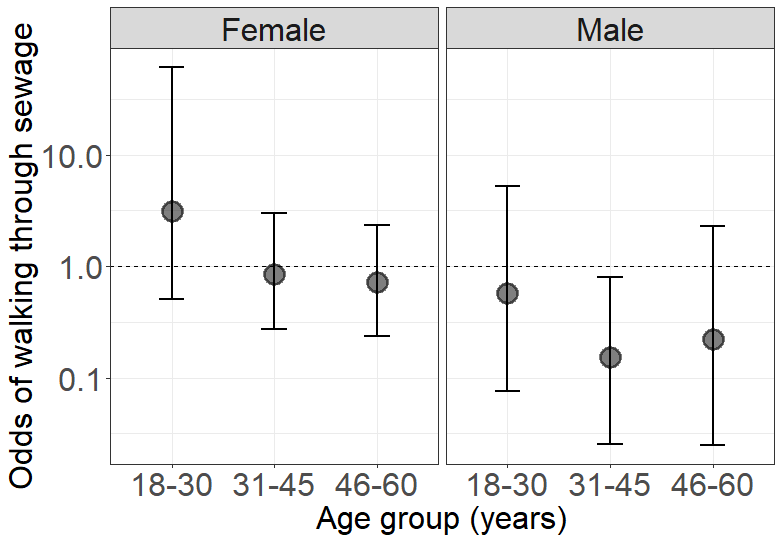

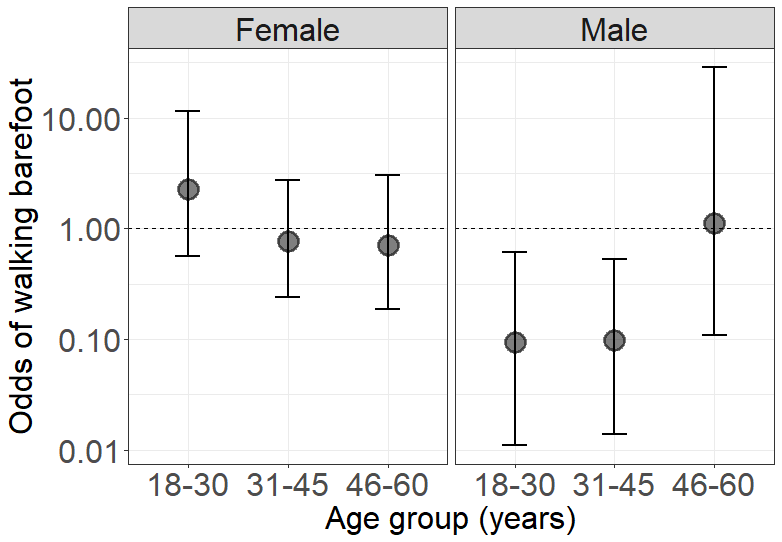

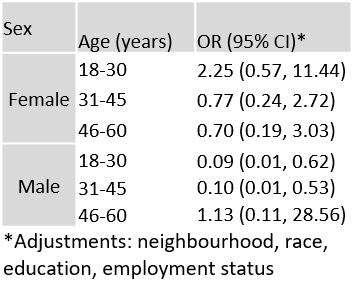

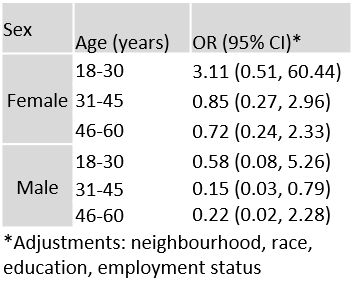
**

**A**

**B**
