# Supplementary material for: Gender differences in Leptospira exposure risk, perceptions of disease severity, and high-risk behaviours in Salvador, Brazil: A cross-sectional study
Source: PLOS Glob Public Health. 2025 Jun 27;5(6):e0004786. doi: 10.1371/journal.pgph.0004786 (PMC12204547; doi:10.1371/journal.pgph.0004786)
Supplement: S1 Table — (DOCX) [file pgph.0004786.s006.docx]

S1 Table: Sex-disaggregated descriptive analysis of seroprevalence across risk factors.

|  | **Females** | | **Males** | |
| --- | --- | --- | --- | --- |
|  | **Total** | **Seroprevalence (%) (n)** | **Total** | **Seroprevalence (%) (n)** |
| **Sociodemographic** |  |  |  |  |
| Age group (years) | 481 |  | 280 |  |
| 18-30 |  | 6.5 (8) |  | 6.5 (5) |
| 31-45 |  | 8.9 (15) |  | 12.9 (13) |
| 46-60 |  | 10.0 (12) |  | 18.3 (13) |
| >60 |  | 10 (14.7) |  | 33.3 (10) |
| Race | 481 |  | 280 |  |
| Black |  | 9.1 (22) |  | 15.9 (23) |
| Other |  | 12.1 (4) |  | 6.7 (1) |
| Pardo |  | 9,2 (19) |  | 14.2 (17) |
| Highest level of school studied | 481 |  | 280 |  |
| Secondary school |  | 5.9 (6) |  | 10.2 (6) |
| Primary school |  | 10.3 (39) |  | 15.8 (35) |
| Employment status (as of previous week) | 480 |  | 279 |  |
| Unemployed |  | 9.8 (27) |  | 20.2 (19) |
| Formal |  | 9.2 (6) |  | 12.8 (12) |
| Informal |  | 8.6 (12)) |  | 11.0 (10) |
| Occupation | 480 |  | 279 |  |
| High-risk |  | 8.2 (4) |  | 21.7 (10) |
| Other |  | 9.0 (14) |  | 8.6 (12) |
| Unemployed |  | 9.8 (27) |  | 20.2 (19) |
| **Perceptions of leptospirosis** |  |  |  |  |
| Perceived severity of leptospirosis | 474 |  | 272 |  |
| Less serious |  | 14.3 (9) |  | 26.5 (9) |
| Extremely serious |  | 8.8 (36) |  | 13.0 (31) |
| **Behaviours (in last 6 months)** |  |  |  |  |
| Walked through flood water | 474 |  | 272 |  |
| Rarely |  | 10.1 (35) |  | 15.1 (32) |
| Frequently |  | 7.9 (10) |  | 15.0 (9) |
| Walked through sewage water | 474 |  | 273 |  |
| Rarely |  | 10.2 (38) |  | 14.6 (31) |
| Frequently |  | 7.0 (7) |  | 16.4 (10) |
| Could wear boots during flooding | 475 |  | 273 |  |
| No |  | 9.5 (37) |  | 15.3 (21) |
| Yes |  | 9.3 (8) |  | 14.7 (20) |
| Walked barefoot | 474 |  | 273 |  |
| Rarely |  | 9.0 (33) |  | 14.0 (29) |
| Frequently |  | 11.1 (12) |  | 18.2 (12) |
| Walked through mud | 475 |  | 273 |  |
| Rarely |  | 9.0 (32) |  | 14.8 (29) |
| Frequently |  | 10.8 (13) |  | 15.6 (12) |
