# Supplementary material for: Gender differences in Leptospira exposure risk, perceptions of disease severity, and high-risk behaviours in Salvador, Brazil: A cross-sectional study
Source: PLOS Glob Public Health. 2025 Jun 27;5(6):e0004786. doi: 10.1371/journal.pgph.0004786 (PMC12204547; doi:10.1371/journal.pgph.0004786)
Supplement: S2 Table — (DOCX) [file pgph.0004786.s007.docx]

S2 Table: Sex-disaggregated univariable logistic regression analysis of seroprevalence across risk factors.

|  | **Combined** | | | **Sex-disaggregated** | | | | | | |
| --- | --- | --- | --- | --- | --- | --- | --- | --- | --- | --- |
|  |  |  |  | Female-restricted | | | Male-restricted | | | |
| **Exposure** | n | OR (95% CI) | *p*-value | n | OR (95% CI) | *p*-value | n | OR (95% CI) | *p*-value |  |
| **Sociodemographic Indictors** |  |  |  |  |  |  |  |  |  |  |
| Gender | 761 |  |  |  |  |  |  |  |  |  |
| Female |  | REF | REF |  | N/A |  |  | N/A |  |  |
| Male |  | 1.66 (1.06, 2.61) | 0.027 |  |  |  |  |  |  |  |
| Age (increase per year) | 761 | 1.02 (1.01, 1.04) | 0.001 | 481 | 1.02 (1.00, 1.05) | 0.12 | 280 | 1.04 (1.01, 1.06) | 0.002 |  |
| Race | 761 |  |  | 481 |  |  | 280 |  |  |  |
| Black |  | REF | REF |  | REF | REF |  | REF | REF |  |
| Other |  | 0.88 (0.33, 2.34) |  |  | 1.45 (0.34, 6.14) | 0.6 |  | 0.38 (0.05, 3.02) | 0.4 |  |
| Pardo |  | 0.94 (0.59, 1.49) | 0.8 |  | 1.00 (0.45, 2.22) | >0.9 |  | 0.88 (0.44, 1.73) | 0.7 |  |
| Highest level of school studied | 761 |  |  | 481 |  |  | 280 |  |  |  |
| Secondary school |  | REF | REF |  | REF | REF |  | REF | REF |  |
| Primary school |  | 1.75 (0.92, 3.30) | 0.086 |  | 2.01 (0.68, 5.96) | 0.2 |  | 1.66 (0.66, 4.16) | 0.3 |  |
| Employment status (in the last week) | 759 |  |  | 480 |  |  | 279 |  |  |  |
| Formally employed |  | REF | REF |  | REF | REF |  | REF | REF |  |
| Unemployed |  | 1.11 (0.62, 1.99) | 0.7 |  | 1.06 (0.33, 3.39) | >0.9 |  | 1.73 (0.79, 3.81) | 0.2 |  |
| Informally employed |  | 0.83 (0.43, 1.60) | 0.6 |  | 0.86 (0.23, 3.15) | 0.8 |  | 0.84 (0.35, 2.06) | 0.7 |  |
| Occupation (among employed) | 389 |  |  | 204 |  |  | 185 |  |  |  |
| Other |  | REF | REF |  | REF | REF |  | REF | REF |  |
| High-risk |  | 1.78 (0.89, 3.57) | 0.1 |  | 0.90 (0.24, 2.64) | 0.9 |  | 3.50 (0.41, 29.6) | 0.3 |  |
| **Perceptions of leptospirosis** | | |  |  |  |  |  |  |  |  |
| Perceived severity of leptospirosis | 746 |  |  | 474 |  |  | 272 |  |  |  |
| Less serious |  | REF | REF |  | REF | REF |  | REF | REF |  |
| Extremely serious |  | 0.51 (0.29, 0.89) | 0.019 |  | 0.54 (0.20, 1.47) | 0.2 |  | 0.42 (0.18, 0.97) | 0.043 |  |
| **Behaviours in the last 6 months** | | | |  |  |  |  |  |  |  |
| Walked through floodwater | 746 |  |  | 474 |  |  | 272 |  |  |  |
| Rarely or never |  | REF | REF |  | REF | REF |  | REF | REF |  |
| Frequently |  | 0.84 (0.49, 1.43) | 0.5 |  | 0.71 (0.26, 1.89) | 0.5 |  | 0.99 (0.45, 2.21) | >0.9 |  |
| Walked through sewage water | 747 |  |  | 474 |  |  | 273 |  |  |  |
| Rarely or never |  | REF | REF |  | REF | REF |  | REF | REF |  |
| Frequently |  | 0.88 (0.50, 1.55) | 0.7 |  | 0.55 (0.17, 1.78) | 0.3 |  | 1.14 (0.53, 2.49) | 0.7 |  |
| Could wear boots during flooding | 748 |  |  | 475 |  |  | 273 |  |  |  |
| Yes |  | REF | REF |  | REF | REF |  | REF | REF |  |
| No |  | 0.86 (0.53, 1.39) | 0.5 |  | 1.02 (0.36, 2.90) | >0.9 |  | 1.05 (0.54, 2.04) | 0.9 |  |
| Walked barefoot outside of home | 747 |  |  | 474 |  |  | 273 |  |  |  |
| Rarely or never |  | REF | REF |  | REF | REF |  | REF | REF |  |
| Frequently |  | 1.32 (0.80, 2.19) | 0.3 |  | 1.41 (0.54, 3.68) | 0.5 |  | 1.36 (0.65, 2.85) | 0.4 |  |
| Walked through mud | 748 |  |  | 475 |  |  | 273 |  |  |  |
| Rarely or never |  | REF | REF |  | REF | REF |  | REF | REF |  |
| Frequently |  | 1.17 (0.71, 1.92) | 0.5 |  | 1.31 (0.53, 3.27) | 0.6 |  | 1.06 (0.51, 2.21) | 0.9 |  |

REF: Reference group.
