# Supplementary material for: Gender differences in Leptospira exposure risk, perceptions of disease severity, and high-risk behaviours in Salvador, Brazil: A cross-sectional study
Source: PLOS Glob Public Health. 2025 Jun 27;5(6):e0004786. doi: 10.1371/journal.pgph.0004786 (PMC12204547; doi:10.1371/journal.pgph.0004786)
Supplement: S3 Table — (DOCX) [file pgph.0004786.s008.docx]

S3 Table: Descriptive analysis of prevalence of behaviours across perceived severity.

| **Behaviour (in last 6 months)** | **Female n, (%)** | | | **Male n, (%)** | | |
| --- | --- | --- | --- | --- | --- | --- |
|  | **n** | **Less serious  n=63** | **Extremely serious  n = 411** | **n** | **Less serious  n=38** | **Extremely serious  n = 238** |
| Walked through flood water | 474 |  |  | 271 |  |  |
| Rarely |  | 45 (71.4) | 303 (73.7) |  | 28 (82.4) | 184 (77.6) |
| Frequently |  | 18 (28.6) | 108 (26.3) |  | 6 (17.6) | 53 (22.4) |
| Walked through sewage water | 474 |  |  | 272 |  |  |
| Rarely |  | 48 (76.2) | 326 (79.3) |  | 24 (70.6) | 188 (79.0) |
| Frequently |  | 15 (23.8) | 85 (20.7) |  | 10 (29.4) | 50 (21.0) |
| Could wear boots during flooding | 474 |  |  | 272 |  |  |
| No |  | 11 (17.5) | 75 (18.2) |  | 15 (44.1) | 121 (50.8) |
| Yes |  | 52 (82.5) | 336 (81.8) |  | 19 (55.9) | 117 (49.2) |
| Walked barefoot | 474 |  |  | 272 |  |  |
| Rarely |  | 50 (79.4) | 316 (76.9) |  | 20 (58.8) | 187 (78.6) |
| Frequently |  | 13 (20.6) | 95 (23.1) |  | 14 (41.2) | 51 (21.4) |
| Walked through mud | 474 |  |  | 272 |  |  |
| Rarely |  | 44 (69.8) | 310 (75.4) |  | 26 (76.5) | 170 (71.4) |
| Frequently |  | 19 (30.2) | 101 (24.6) |  | 8 (23.5) | 68 (28.6) |
