# Supplementary material for: Gender differences in Leptospira exposure risk, perceptions of disease severity, and high-risk behaviours in Salvador, Brazil: A cross-sectional study
Source: PLOS Glob Public Health. 2025 Jun 27;5(6):e0004786. doi: 10.1371/journal.pgph.0004786 (PMC12204547; doi:10.1371/journal.pgph.0004786)
Supplement: S4 Table — (DOCX) [file pgph.0004786.s009.docx]

S4 Table: Sex-disaggregated univariable logistic regression analysis of the association of perceived severity with high-risk behaviours.

| **Perceived severity of leptospirosis** | **Behaviour** | | | | | | | | | | | | | | |
| --- | --- | --- | --- | --- | --- | --- | --- | --- | --- | --- | --- | --- | --- | --- | --- |
|  | Walked through flood water | | | Walked through sewage water | | | Could wear boots during flooding | | | Walked barefoot | | | Walked through mud | | |
|  | n | OR  (95% CI) | *p* | n | OR  (95% CI) | *p* | n | OR  (95% CI) | *p* | n | OR  (95% CI) | *p* | n | OR  (95% CI) | *p* |
| **Combined** | 745 |  |  | 272 |  |  | 746 |  |  | 746 |  |  | 272 |  |  |
| Less serious |  | REF |  |  | REF |  |  | REF |  |  | REF |  |  | REF |  |
| Extremely serious |  | 1.01  (0.53, 1.86) | >0.9 |  | 0.70  (0.39, 1.24) | 0.2 |  | 0.85  (0.49, 1.48) | 0.6 |  | 0.73  (0.42, 1.25) | 0.2 |  | 0.73  (0.25, 2.11) | 0.6 |
| **Sex-disaggregated** | | | | | | | | | | | | | | | |
| Female-restricted | 474 |  |  | 474 |  |  | 474 |  |  | 474 |  |  | 474 |  |  |
| Less serious |  | REF |  |  | REF |  |  | REF |  |  | REF |  |  | REF |  |
| Extremely serious |  | 0.83  (0.36, 1.93) | 0.7 |  | 0.70  (0.29, 1.69) | 0.4 |  | 0.95  (0.47, 1.90) | 0.9 |  | 1.23  (0.57, 2.67) | 0.6 |  | 0.64  (0.28, 1.48) | 0.3 |
| Male-restricted | 271 |  |  | 272 |  |  | 272 |  |  | 272 |  |  | 272 |  |  |
| Less serious |  | REF |  |  | REF |  |  | REF |  |  | REF |  |  | REF |  |
| Extremely serious |  | 1.51  (0.38, 6.03) | 0.6 |  | 0.61  (0.24, 1.52) | 0.3 |  | 0.71  (0.25, 2.01) | 0.5 |  | 0.39  (0.18, 0.82) | 0.014 |  | 1.41  (0.47, 4.28) | 0.5 |

REF: Reference group.
