# Supplementary material for: Gender differences in Leptospira exposure risk, perceptions of disease severity, and high-risk behaviours in Salvador, Brazil: A cross-sectional study
Source: PLOS Glob Public Health. 2025 Jun 27;5(6):e0004786. doi: 10.1371/journal.pgph.0004786 (PMC12204547; doi:10.1371/journal.pgph.0004786)
Supplement: S5 Table — (DOCX) [file pgph.0004786.s010.docx]

S5 Table: Total causal effect estimates for the effect of perceived severity on the risk of performing high-risk behaviours, shown for the combined and sex-disaggregated multivariable logistic regression models.

| **Perceived severity of leptospirosis** | **Behaviour** | | | | | | | | | | | | | | |
| --- | --- | --- | --- | --- | --- | --- | --- | --- | --- | --- | --- | --- | --- | --- | --- |
|  | Walked through  flood water | | | Walked through  sewage water | | | Could wear boots  during flooding | | | Walked barefoot outside the home | | | Walked through  mud | | |
|  | n | aOR  (95% CI)* | *p* | n | aOR  (95% CI)* | *p* | n | aOR  (95% CI)** | *p* | n | aOR  (95% CI)*** | *p* | n | aOR  (95% CI)* | *p* |
| **Combined** | 724 |  |  | 725 |  |  | 725 |  |  | 726 |  |  | 725 |  |  |
| Less serious |  | REF |  |  | REF |  |  | REF |  |  | REF |  |  | REF |  |
| Extremely serious |  | 0.96  (0.45, 1.67) | >0.9 |  | 0.67  (0.40, 1.15) | 0.13 |  | 0.84  (0.44, 1.60) | 0.6 |  | 0.64  (0.37, 1.13) | 0.12 |  | 0.79  (0.45, 1.38) | 0.4 |
| **Sex-disaggregated** | | | | | | | | | | | | | | |  |
| Female-restricted | 462 |  |  | 462 |  |  | 462 |  |  | 463 |  |  | 462 |  |  |
| Less serious |  | REF |  |  | REF |  |  | REF |  |  | REF |  |  | REF |  |
| Extremely serious |  | 0.84  (0.38, 1.86) | 0.7 |  | 0.80  (0.41, 1.62) | 0.5 |  | 0.87  (0.42, 1.81) | 0.7 |  | 1.15  (0.53, 2.50) | 0.7 |  | 0.76  (0.37, 1.58) | 0.5 |
| Male-restricted | 262 |  |  | 263 |  |  | 263 |  |  | 263 |  |  | 263 |  |  |
| Less serious |  | REF |  |  | REF |  |  | REF |  |  | REF |  |  | REF |  |
| Extremely serious |  | 1.00  (0.32, 3.11) | >0.9 |  | 0.41  (0.17, 1.00) | 0.049 |  | 0.75  (0.24, 2.63) | 0.7 |  | 0.24  (0.08, 0.76) | 0.015 |  | 0.83  (0.26, 2.66) | 0.6 |
| Test for interaction |  |  |  | 0.7 |  | 0.3 |  |  | 0.4 |  |  | 0.007 |  |  | 0.4 |
| REF: Reference group. Adjustments: *neighbourhood, age, gender, race, food insecurity, employment, education; **age, gender, race, food insecurity, employment, education; ***neighbourhood, age, gender, race, food insecurity, employment, education. Gender was removed as a confounder in the sex-disaggregated models. | | | | | | | | | | | | | | | |
