# Supplementary material for: Gender differences in Leptospira exposure risk, perceptions of disease severity, and high-risk behaviours in Salvador, Brazil: A cross-sectional study
Source: PLOS Glob Public Health. 2025 Jun 27;5(6):e0004786. doi: 10.1371/journal.pgph.0004786 (PMC12204547; doi:10.1371/journal.pgph.0004786)
Supplement: S6 Table — (DOCX) [file pgph.0004786.s011.docx]

**S6 Table: Descriptive analysis of age across explanatory variables in men (A) and women (B)**

**(A)**

| **Characteristic** | **Age group (years)** | | | | **p-value***^2^* |
| --- | --- | --- | --- | --- | --- |
|  | **18-30** | **31-45** | **46-60** | **>60** |  |
|  | N = 78*^1^* | N = 101*^1^* | N = 71*^1^* | N = 30*^1^* |  |
| Perceived severity of leptospirosis | | | | | 0.12 |
| Less serious | 9 (12.7%) | 10 (9.9%) | 7 (10.0%) | 8 (26.7%) |  |
| Extremely serious | 62 (87.3%) | 91 (90.1%) | 63 (90.0%) | 22 (73.3%) |  |
| **Behaviours (in the last 6 months)** | | | | | |
| Walked through sewage water | | | | | 0.4 |
| Rarely | 55 (77.5%) | 74 (73.3%) | 57 (80.3%) | 26 (86.7%) |  |
| Frequently | 16 (22.5%) | 27 (26.7%) | 14 (19.7%) | 4 (13.3%) |  |
| Walked barefoot |  |  |  |  | 0.017 |
| Rarely | 44 (62.0%) | 81 (80.2%) | 57 (80.3%) | 25 (83.3%) |  |
| Frequently | 27 (38.0%) | 20 (19.8%) | 14 (19.7%) | 5 (16.7%) |  |
| *^1^* n (%) | | | | | |
| *^2^* Fisher’s exact test; Pearson’s Chi-squared test | | | | | |

**(B)**

| **Characteristic** | **Age group (years)** | | | | **p-value***^2^* |
| --- | --- | --- | --- | --- | --- |
|  | **18-30** | **31-45** | **46-60** | **>60** |  |
|  | N = 124*^1^* | N = 169*^1^* | N = 120*^1^* | N = 68*^1^* |  |
| Perceived severity of leptospirosis | | | | | 0.2 |
| Less serious | 11 (9.2%) | 20 (11.9%) | 22 (18.5%) | 10 (14.7%) |  |
| Extremely serious | 108 (90.8%) | 148 (88.1%) | 97 (81.5%) | 58 (85.3%) |  |
| **Behaviours (in the last 6 months)** | | | | | |
| Walked through sewage water | | | | | 0.003 |
| Rarely | 96 (80.7%) | 122 (72.6%) | 92 (77.3%) | 64 (94.1%) |  |
| Frequently | 23 (19.3%) | 46 (27.4%) | 27 (22.7%) | 4 (5.9%) |  |
| Walked barefoot |  |  |  |  | <0.001 |
| Rarely | 73 (61.3%) | 127 (75.6%) | 102 (85.7%) | 64 (94.1%) |  |
| Frequently | 46 (38.7%) | 41 (24.4%) | 17 (14.3%) | 4 (5.9%) |  |
| *^1^* n (%) | | | | | |
| *^2^* Pearson’s Chi-squared test | | | | | |
