# Supplementary material for: Gender differences in Leptospira exposure risk, perceptions of disease severity, and high-risk behaviours in Salvador, Brazil: A cross-sectional study
Source: PLOS Glob Public Health. 2025 Jun 27;5(6):e0004786. doi: 10.1371/journal.pgph.0004786 (PMC12204547; doi:10.1371/journal.pgph.0004786)
Supplement: Attachment — Submitted filename: Response to reviewer and journal comments_gender lepto.docx [file pgph.0004786.s014.docx]

Contents

[Response to journal comments	1](#_Toc184820722)

[Response to reviewer comments	3](#_Toc184820723)

[Reviewer 1	3](#_Toc184820724)

[Reviewer 2	4](#_Toc184820725)

[Responses to annotated PDF from reviewer 2	5](#_Toc184820726)

We thank the journal and reviewers for their constructive comments and the time and effort they invested in the review. We have carefully considered the feedback and made edits to the manuscript, which have enhanced its quality. In this response document, we address both the journal's and reviewers' comments, referencing line numbers where changes have been made which corresponds to the tracked-changes version of the manuscript. Additionally, for Reviewer 2's feedback which was provided in an annotated PDF, we have referenced the page number from the PDF document.

We would like to clarify the issue regarding access to the dataset and scripts for the analyses. The dataset and associated scripts have been uploaded to the Open Science Framework (OSF), and [the link](https://osf.io/ebdtj/?view_only=a0e165ca71f14e2ab1985b730b5dfac5) provided on the editorial manager has been checked. It is functioning correctly on our end, and we have been able to access both the data and code without any issues. We would kindly request further clarification on the specific problem or error encountered, as we are currently unable to identify any issues with the link. Additionally, we would like to note that the code has recently been updated due to the inclusion of a new analysis, though this update is accessible through the same OSF link provided earlier

# Response to journal comments

| Comment | Response |
| --- | --- |
| 1. Please include a complete copy of PLOS’ questionnaire on inclusivity in global research in your revised manuscript. Our policy for research in this area aims to improve transparency in the reporting of research performed outside of researchers’ own country or community. The policy applies to researchers who have travelled to a different country to conduct research, research with Indigenous populations or their lands, and research on cultural artefacts. The questionnaire can also be requested at the journal’s discretion for any other submissions, even if these conditions are not met.  Please find more information on the policy and a link to download a blank copy of the questionnaire here: https://journals.plos.org/globalpublichealth/s/best-practices-in-research-reporting. Please upload a completed version of your questionnaire as Supporting Information when you resubmit your manuscript. | The PLOS Inclusivity in Global Research has been uploaded in the supporting information document. |
| 2. Please amend your detailed Financial Disclosure statement. This is published with the article. It must therefore be completed in full sentences and contain the exact wording you wish to be published.  1. Please clarify all sources of funding (financial or material support) for your study. List the grants (with grant number) or organizations (with url) that supported your study, including funding received from your institution.  2. State the initials, alongside each funding source, of each author to receive each grant. 3. State what role the funders took in the study. If the funders had no role in your study, please state: “The funders had no role in study design, data collection and analysis, decision to publish, or preparation of the manuscript.” 4. If any authors received a salary from any of your funders, please state which authors and which funders.   If you did not receive any funding for this study, please simply state: “The authors received no specific funding for this work.” | The detailed Financial Disclosure statement has been updated in the manuscript and on the editorial manager. |
| 3. Please send a completed 'Competing Interests' statement, including any COIs declared by your co-authors. If you have no competing interests to declare, please state "The authors have declared that no competing interests exist". Otherwise please declare all competing interests beginning with the statement "I have read the journal's policy and the authors of this manuscript have the following competing interests:" | A competing interests statement has been added to the submission in the supporting information as a word document, this has also been updated on the editorial manager. |
| 4. "Figure 3": Please confirm whether you drew the images / clip-art within the figure panels by hand. If you did not draw the images, please provide (a) a link to the source of the images or icons and their license / terms of use; or (b) written permission from the copyright holder to publish the images or icons under our CC-BY 4.0 license. Alternatively, you may replace the images with open source alternatives. See these open source resources you may use to replace images / clip-art: - https://commons.wikimedia.org, - https://openclipart.org/ | The figure was produced in Microsoft PowerPoint, where the icons were also located. These icons are free to use and there is no royalty or copyright associated, as can be seen [via this link](https://support.microsoft.com/en-gb/office/insert-icons-in-microsoft-365-e2459f17-3996-4795-996e-b9a13486fa79#:~:text=Next%3A%20Present%20slideshows-,Insert%20icons%20in%20Microsoft%20365,or%20copyright%20to%20worry%20about.). |
| 5. "Figure 1": please (a) provide a direct link to the base layer of the map (i.e., the country or region border shape) and ensure this is also included in the figure legend; and (b) provide a link to the terms of use / license information for the base layer image or shapefile. We cannot publish proprietary or copyrighted maps (e.g. Google Maps, Mapquest) and the terms of use for your map base layer must be compatible with our CC-BY 4.0 license.  Note: if you created the map in a software program like R or ArcGIS, please locate and indicate the source of the basemap shapefile onto which data has been plotted. If your map was obtained from a copyrighted source please amend the figure so that the base map used is from an openly available source. Alternatively, please provide explicit written permission from the copyright holder granting you the right to publish the material under our CC-BY 4.0 license.   Please note that the following CC BY licenses are compatible with PLOS license: CC BY 4.0, CC BY 2.0 and CC BY 3.0, meanwhile such licenses as CC BY-ND 3.0 and others are not compatible due to additional restrictions.    If you are unsure whether you can use a map or not, please do reach out and we will be able to help you. The following websites are good examples of where you can source open access or public domain maps:  * U.S. Geological Survey (USGS) - All maps are in the public domain. (http://www.usgs.gov)  * PlaniGlobe - All maps are published under a Creative Commons license so please cite “PlaniGlobe, http://www.planiglobe.com, CC BY 2.0” in the image credit after the caption. (http://www.planiglobe.com/?lang=enl)  * Natural Earth - All maps are public domain. (http://www.naturalearthdata.com/about/terms-of-use/) | The Salvador boarder is sourced from GADM and the satellite imagery has been sourced from Maxar, Satellite WorldView-3 on 15/04/2022. These sources have been added to the figure legend.  GADM is open source as can be [seen here](https://gadm.org/license.html#google_vignette):  The satellite imagery can be published, as can be [seen here](https://www.maxar.com/legal/display-media-license) or from the exert below::  **“GRANT OF LICENSE.** In addition to those use rights set forth in any other license granted by Maxar covering the Licensed Images, Maxar grants to Customer a perpetual, non-exclusive, non-transferable license to publish extracts of the Licensed Images and/or Derivatives of the Licensed Images in any of the following publications (the “Publications”) to supplement text or other content, subject to the requirements set forth in Section 2 below:  (a) reports, white papers, presentations and training manuals (print and electronic);  (b) books, textbooks, newspapers, magazines, articles, journals and blogs (print and electronic);  (c) posters, brochures and booth displays;  (d) websites and social media; and  (e) video media including movies, television programs” |

# Response to reviewer comments

## Reviewer 1

| Comment | Response |
| --- | --- |
| From the hypothesis to the final results, the study "Gender differences in the perception of leptospirosis severity, behaviours, and Leptospira exposure risk in urban Brazil" shows how gender affects (cultural) perception and risk behaviour among humans and the effects of this on the exposure risk and severity of diseases. This is significant for health education, and generally preventive medicine/care. The study design is a cross sectional study which involved some parts of Brazil. However, the researchers failed to exclusively state how they came about the sample size of 761. Also, with regards to the in-text citation: for Vancouver style, the reference number is placed to the left of or inside of colons and semi-colons; but placed to the right of or outside of full stop. | Thank you for your time and effort in reviewing our manuscript.  We appreciate your feedback regarding the clarification of the sample size. To arrive at the sample size of 761 we first identified eligible residents living in areas of high-risk through a previous census. Of the 1,185 residents eligible, 269 were in households that did not give household consent, and 147 individuals declined. Of the 769 residents who consented, 8 were excluded as they serostatus data were missing. This resulted in 761 individuals included in the study.  The sampling method has been clarified in the methods section from lines 167, and “resulting in 761 individuals analysed in this study” has been added to the sentence “Of these 916 residents, 147 (16%) declined to participate and 8 (1%) were excluded from this analysis due to undetermined serostatus, resulting in 761 individuals included in this study.” on line 354.  Regarding in-text citations, we have followed the PLOS GPH guidelines [found here](https://journals.plos.org/globalpublichealth/s/submission-guidelines#:~:text=References%20are%20listed%20at%20the,to%20analyze%20the%20data%E2%80%9D).), which specify citing reference numbers in square brackets (e.g., "We used the techniques developed by our colleagues [19] to analyze the data"). Accordingly, we have maintained the previous citation style, replacing round brackets with square brackets. |

## Reviewer 2

| Comment | Response |
| --- | --- |
| This is an interesting article, though its scope appears to be more locally focused. I believe it would benefit from including recommendations that bridge academia with health services, public health surveillance, and strategies for community empowerment and participation in health.  Several points could benefit from further elaboration. While the methodology is comprehensive, it could be made more concise. Additionally, I felt that more information on the sampling aspects of the study would enhance the clarity of the methodology.  Further detailed comments can be found in the attached document. | Thank you for your time and effort in reviewing this manuscript. Your constructive feedback has been invaluable in enhancing its quality.  In response to your recommendation regarding public health services, we have added the following on line 652: *“Community dissemination of these findings is important, and organisations like the Centre for the Control of Zoonoses (CCZ) could play a key role in exploring the influence of perceptions, as their health agents provide door-to-door health advice.”*  We have elaborated on various points raised in the annotated PDF, including sampling methods (from line 184), limitations (from line 598), and conclusions (from line 637). Additionally, we conducted a sub-analysis of high-risk behaviours by gender and age group, as per your insightful suggestion. This analysis revealed distinct patterns across age groups, which are discussed in a subsequent comment. The inclusion of this sub-analysis required updates to the methodology section, starting at line 322. Combined with the expanded explanation of sampling techniques, the methodology remains extensive; however, we believe this level of detail is warranted to support the analysis.  Further responses to the comments from the annotated PDF have been copied into the table below. |

### Responses to annotated PDF from reviewer 2

Page number (Pg) corresponds to the annotated PDF document that the comments were received in.

Due to the formatting of the comments received, we have been unable to respond to all of the comments – i.e., on occasion where certain sections were highlighted but there were no supporting comments. We have indicated these in the table below with “We have been unable to address this comment as we weren't sure what the annotation was responding to. Kindly clarify if we have overlooked anything.” Please note that this has only been for 8 out of 92 annotations across the manuscript and we feel the comments and response have been otherwise comprehensive.

| **Pg** | **Comment** | **Response** |
| --- | --- | --- |
| 1 | An interesting article. Local made, not wide. Useful fir local health services. Would be better used in Brazilian journals such as ciencia e saúde coletiva ou cadernos de saúde publica | Thank you for the thoughtful comment. We believe the article is well-suited to PLOS Global Public Health due to its focus on gender-based differences, an area currently understudied not only in leptospirosis but across other zoonotic diseases. Community-based research studies like this one (which collects data on individuals living in four different communities) are essential for understanding how zoonoses and climate are likely to affect health and are widely published in PLOS GPH for this reason. Publishing in PLOS GPH enables us to reach a global audience, allowing for broader dissemination of our methods. This, in turn, could support replication of our approach in research on other zoonoses and climate-sensitive infectious diseases. |
| 1 | In a Brazilian capital. Using "urban brazil" transmits an idea that the entire urban area of Brazil is being analyzed. And this is misleading. Also, insert the year of the cross-sectional survey in the title | Thank you for this insightful comment. We have amended the title to "Gender differences in Leptospira exposure risk, perceptions of disease severity, and high-risk behaviours in Salvador, Brazil: a cross-sectional study (2021-2022)" |
| 1 | Insert keyword: public health | Inserted keyword on line 76. |
| 1 | This is not gender, it's age. | We have removed "boys, girls" from the sentence on line 51. |
| 1 | Study's objective is missing | The objective has been added to the abstract on lines 56-57. |
| 1 | Separate this topics | The topics headers (e.g., methodology/key findings) have been removed as per PLOS GPH requirements, however the methodology and results paragraphs have been separated. |
| 1 | Inset information on sampling methods and data analysis methods used | Information on how communities were selected (from previously collected surveillance data) has been added on line 60. Additionally, logistic regression modelling has been added on line 61 to briefly describe data analysis methods. |
| 1 | Insert gender percentages | The number of men and women included in the study has been added to line 59. |
| 1 | Insert Ci95% | Seroprevalence 95% CIs across men and women has been added to line 65. |
| 1 | Insert OR | ORs of key results has been added starting from line 67. |
| 1 | Where? I hope there is a good characterization of the environment | Thank you for identifying the need for clarification here; the variable walking barefoot refers to outside of the home. This is described in the main methods section, but has now been clarified in the abstract section on line 68. |
| 1 | Who are the group 1 and 2 for the cross sectional analysis? | The reference group changes per each generalised linear model (e.g., men who don't walk barefoot outside the home compared to men who do walk barefoot outside of the home). |
| 2 | Nice. How you suggest to do that? Who is responsible? The universities? Health services? SES-BA ? ISC-UFBA? CONASEMS? COSEMS? POPULAR initiatives? Who? | Thank you for your suggestions to highlight the key actors to communicate these findings to. We have added a sentence on line 652 in the discussion to reflect this: 'Community dissemination of these findings is important, and organisations like the Centre for the Control of Zoonoses (CCZ) could play a key role in exploring the influence of perceptions as their health agents provide door-to-door health advice.' |
| 2 | Blank | The link to the data works for us. Kindly clarify what this comment is responding to if we have misunderstood. |
| 4 | For parallelism, use English here | Thank you for spotting this mistake, the affiliation has been changed to English. |
| 4 | A fundacao Osvaldo Cruz não faz parte do Ministério da Saúde. Hà algum engano Nesta afiliacao (The Osvaldo Cruz foundation is not part of the Ministry of Health. There is some mistake in this affiliation ) | This mistake in the affiliation has been amended to "Moniz Gonçalo Moniz Institute, Oswaldo Cruz Foundation, Salvador, Bahia, Brazil" to line 17. Thank you for identifying. |
| 7 | Insert reference | Two references have been added on line 108 to support the sentence that "Whilst previous studies have investigated the odds of one sex having a higher risk of exposures or *Leptospira* seropositivity compared to another sex". These references are https://journals.plos.org/plosntds/article?id=10.1371/journal.pntd.0009256 and https://journals.plos.org/globalpublichealth/article?id=10.1371/journal.pgph.0000408. Both studies discuss gendered differences in odds of *Leptospira seropositivity or high-risk behaviour.* |
| 8 | Blank | We have been unable to address this comment as we weren't sure what the annotation was responding to. Kindly clarify if we have overlooked anything. |
| 8 | What is the reference for that? Please, include | To support the statement "climate change continues to expose marginalised individuals to CSIDs and embed gendered inequalities", four references have been added across a review of climate sensitive infectious diseases and IPCC reports: https://www.nature.com/articles/s41558-022-01426-1, https://www.ipcc.ch/report/ar6/wg2/chapter/summary-for-policymakers/, https://www.ipcc.ch/report/ar6/wg2/ and from: https://www.ipcc.ch/report/ar6/syr/. |
| 9 | Where is the study design? Cross-sectional? Initiate with it. Insert info on the conduction of a survey, and year of the study | The "Study design" header was a sub-header to "Study area", and "Questionnaires and serosurveys" etc, which is why it may have appeared to be missing as no text directly followed it. This has been amended so that a sentence to describe the cross-sectional study design and the year of study has been added immediately after "Study design", from line 158. |
| 9 | Relocate it | "In Salvador" has been added to the study design (as previously on line 158) and to the study area on line 162. |
| 9 | Where is the sampling method section? | A paragraph from the "Questionnaires and serosurveys" section has been moved into a newly added "Eligibility and enrolment" section on line 181. This paragraph has been modified to include descriptions of the sampling methods, e.g., more details surrounding the initial census, as suggested in later comments. |
| 9 | Please insert population size from the estimated IBGE census of the survey year. | The estimated population from IBGE, or 2,417,678 in 2022, has been added to the description of the study area on line 162. |
| 9 | Collected how and in which system? | The communities were identified as high risk by surveillance data between 1996-2018 using the Notifiable Diseases Information System (SINAN). This has been added to the methods section on line 165. |
| 9 | How low? 1 minimum wage? Throughout the entire 23 years timeseries? | The average of the residents income per capita is below that of the poverty threshold (half of the minimum wage), as defined by the World Bank. We have calculated this based on the minimum wage of 2022 and have expanded on this explanation in the methods from line 169: “Poverty levels are high, with the average monthly income per capita across communities (R$463) being less than half the minimum wage in 2022 (R$1,212) , as defined by the World Bank's poverty threshold (33).  Reference: International Bank for Reconstruction and Development / The World Bank. Brazil Poverty and Equity Assessment: Looking Ahead of Two Crises. Washington DC; 2022. |
| 9 | Slums? | We have chosen to keep the term informal settlement over slum as we believe this is more appropriate for focusing on the structural challenges. It is a widely used term in the urban health literature that avoids the pejorative term 'slum' which has negative connotations. |
| 9 | Why? ( the answer is obvious I know, but it is good to insert the reason here) | The study sites were defined as areas within 40m of an open sewer to ensure the inclusion of residents living in areas with a higher risk of leptospirosis transmission, this has been added to line 173. |
| 9 | Blank | We have been unable to address this comment as we weren't sure what the annotation was responding to. Kindly clarify if we have overlooked anything. |
| 9 | A census was conducted? It seems really hard to have a census done by the research group. Please, elaborate on this matter. Detail it... personal needed, amount of urveyed participants, etc | A description of the census has been given in the methods from line 183: "An initial census was conducted to enumerate all residents living within the four study areas. The census included all houses located within 40 meters of the open sewer located within each study area and along a 150 – 200 metre stretch of it. The total census counted 1,891 residents living in households within the four areas." |
| 9 | How many? | The census identified 1,891 individuals, this has been added to the methods to line 184. |
| 9 | Why 6 and not 5, or 4? Or 7? | Six months was the minimum period that a resident must have lived in a community to be eligible for inclusion as this was considered as the threshold to accurately represent the epidemiological characteristics of the study population. This has been clarified in the methods section from line 190 as: "A minimum period of six months was established as the threshold to consider an individual as having resided in the area long enough to accurately represent the epidemiological characteristics of the study population.” |
| 9 | What is this 30 number? | This is a reference to the overarching trial protocol. Please note that in the updated manuscript, this is reference 31. |
| 9 | Is this stored in UFBA? | Yes, written consent is stored at UFBA. This has been added to the methods section from line 192 as: "Written informed consent was obtained from each participating individual, consent forms are securely stored at the Federal University of Bahia and will be kept for up to 10 years." |
| 14 | Treated | This has been amended. |
| 15 | Did the authors compare the positives cases epidemiological profile caracteristicas to the epidemiological profile present in SINAN for the region? Are they similar ? | Thank you for this insightful comment. We believe this is outside of the scope of this analysis, however your suggestion raises an important point. We acknowledge that SINAN data, being skewed toward more severe cases, could provide valuable insights, particularly in exploring gender differences. This is an interesting avenue for future analysis, and we will consider it in subsequent studies. |
| 16 | Was there statistical differences between the positive individuals epidemiological profiles and the negative individual ?? | This first table is designed to describe the study population and highlight differences by gender, as this is the primary focus of this analysis. Statistical differences by seroprevalence are addressed in Analysis 1, with a descriptive cross table by seroprevalence provided in Supplementary Information 2a. Statistical differences were formally tested using multivariable logistic regression models, as presented in Table 2.  At the 95% confidence level, the following statistically significant differences were observed among positive individuals: Men: The odds of being seropositive increased by 4% (OR: 1.04, 95% CI: 1.01–1.06) for every additional year of age. Men in high-risk occupations had 3.92 times higher odds of being seropositive (95% CI: 1.17–13.2) compared to men in other occupations. Men who perceived leptospirosis as a serious disease had lower odds (OR: 0.38, 95% CI: 0.15–0.99) of being seropositive than those who perceived it as less serious. No statistically significant differences were observed for women at the 95% confidence level. |
| 16 | This table formatting is not adequate. Lose the margins. Also, lose the "total" for each variable. Add "missimg/unavailable" for each variable | Table 1 has been redone giving missing values where appropriate and to display all reference groups within a variable for clarity |
| 16 | I would like to see this crosstable by seropositivity | Table 1 is intended to describe the study population and differences by gender as this is the focus of this specific analysis. A descriptive cross table by seropositivity is provided as supplementary information 2a and is later analysed in the logistic regression models in Table 2. |
| 16 | No decimal numbers ?? | Table 1 has been amended to give percentages to 1 decimal place |
| 16 | How many years of study is "primary education"? Kindergarten? 4 years? 8 years? 9 years? | The variable name has been changed to "Highest level of school studied" across all relevant tables, and defined in the methods on line 208: "Highest level of school studied was defined as either primary school (including up to 9th grade) or secondary school (above 9th grade)." |
| 16 | I am confused. 370+760+389 is over 759. Is there any typing errors? | Corrected (this was a typo, thank you for identifying) |
| 16 | Employed are : 760+ 389. Correct the table. Something is wrong | Corrected (this was a typo, thank you for identifying) |
| 16 | Blank | p-value placement has been amended. |
| 16 | Blank | p-value placement has been amended. |
| 16 | Lose these internal lines | Internal lines in table 1 have been removed. |
| 16 | Are these pvalues in the write place ?? | Placement has changed with the new table formatting |
| 17 | This is methods section | All GAM analysis results have been moved to the methods section and can be found on lines 276 (Analysis 1) and 315 (Analysis 2) |
| 20 | The comparative group is 'healthy males', right? If so, that's is not so explicit in the text | The reference group changes per each GLM (e.g., men who don't walk barefoot outside the home compared to men who do walk barefoot outside of the home). |
| 20 | Elaborate on the need of this p-value intercation | The inclusion of the p-value for interaction was a key aspect of our analysis to evaluate whether the associations between exposures and the outcome differ by sex. This aligns with the primary objective of our study, which is to investigate differences in risk factors for seropositivity between females and males, and is discussed in the Methods section on line 295: "Finally, the strength of evidence supporting sex as an effect modifier for the relationship between each exposure and outcome pair was investigated by including an interaction term in the multivariable model." |
| 20 | Blank | This refers to the reference group in each model, a footnote clarifying this has been added to the table. |
| 20 | Blank | This refers to the reference group in each model, a footnote clarifying this has been added to the table. |
| 20 | Blank | This refers to the reference group in each model, a footnote clarifying this has been added to the table. |
| 20 | It is not clear to me what are the "groups 2" (or control group, if this vocabulary helps the comprehension of my comment) | The reference group changes per each GLM (e.g., men who don't walk barefoot outside the home compared to men who do walk barefoot outside of the home). |
| 20 | Blank | See response to last comment on page 20. |
| 20 | Bad formating | Corrected |
| 20 | Blank | We have been unable to address this comment as we weren't sure what the annotation was responding to. Kindly clarify if we have overlooked anything. |
| 20 | Blank | See response to last comment on page 20. |
| 20 | Blank | We have been unable to address this comment as we weren't sure what the annotation was responding to. Kindly clarify if we have overlooked anything. |
| 20 | Blank | See response to last comment on page 20. |
| 20 | Lose these 'totals' | Thank you for your suggestion. We have decided to retain the 'totals' column, as the number of people in each model varies slightly, and we believe this is important to show. Since the table already includes numerous footnotes defining the confounders adjusted for in each model, adding additional footnotes to explain missing variables might complicate the presentation. Therefore, we believe including a 'totals' column provides the simplest and clearest solution. |
| 21 | Blank | See response to last comment on page 20. |
| 21 | Blank | We have been unable to address this comment as we weren't sure what the annotation was responding to. Kindly clarify if we have overlooked anything. |
| 21 | Blank | We have been unable to address this comment as we weren't sure what the annotation was responding to. Kindly clarify if we have overlooked anything. |
| 21 | Blank | This was a typo thank you for spotting. We have removed REF from p-value column |
| 21 | Blank | This was a typo thank you for spotting. We have removed REF from p-value column |
| 21 | Blank | We have been unable to address this comment as we weren't sure what the annotation was responding to. Kindly clarify if we have overlooked anything. |
| 21 | Blank | We have been unable to address this comment as we weren't sure what the annotation was responding to. Kindly clarify if we have overlooked anything. |
| 24 | Blank | The first paragraph of the discussion is intended to provide a brief recap of results. Therefore, instead of breaking it into three paragraphs as suggested, we have kept it as a single paragraph but have streamlined it. |
| 25 | Blank | This paragraph, from line 499, provides a brief recap of results to begin the discussion. Therefore, instead of breaking it into three paragraphs as suggested, we have kept it as a single paragraph but have streamlined it. |
| 27 | Insert a limitations section | A limitations sub-title has been inserted on line 593. |
| 27 | The authors should comment on the limitations related to sampling, interviewing techniques, and the inference for the target population | Thank you for the reflective comment. The limitations section from line 593 has been changed and now addresses: Serostatus as an indicator of disease burden, self-reporting of exposures and social-desirability bias during interviewing, sample size issues in the context of smaller number of seropositive women, and treating gender as a binary. |
| 28 | These conclusions are generic and shroud be completely modified. The authors did not consider how the public health services should act based on the results of this research. Also, did not answer the study's objective. So, to help you construct the conclusion, please answer the following questions: 1- how Leptospira infection risk varies among men and women living in the four informal communities in Salvador Brazil? 2-what must be done to change that ? | Thank you for this constructive comment. The conclusion has been redone from line 634, highlighting how *Leptospira* infection risk varies among men and women, discussing potential public health interventions based on this, and suggesting the use of gender-sensitive techniques across other climate-sensitive infectious diseases. |
| 28 | Blank | Corrected |
| 29 | Can't read page number | Line number formatting error corrected, thank you for identifying this mistake. |
| 29 | Blank | Line number formatting error corrected, thank you for identifying this mistake. |
| 30 | Blank | Line number formatting error corrected, thank you for identifying this mistake. |
| 30 | Blank | Line number formatting error corrected, thank you for identifying this mistake. |
| 33 | Outdated | This reference has been updated but uses the original source which would still be considered outdated as published in 2012. We have kept this reference as it is the WHO document used to inform previous KAP studies the group have conducted and provides the theory for the behaviour change model that has not been updated. |
| 34 | Outdated | This reference has not been updated as it is the citation required by REDCap, as found here - https://projectredcap.org/resources/citations/ |
| 34 | Blank | This reference has not been updated as it describes serodiagnosis by microscopic agglutination test which has not changed since 2014 |
| 34 | Blank | Reference updated |
| 35 | Who is the author of the package? | Reference updated |
| 38 | Are there pictures/photos of the area? | Study site photos have been added to figure 1. |
| 39 | Insert legend.... what do the arrows mean? | The legend can be found in the main text on line 244. This formatting style, whereby figures are separated by their legends, are for review purposes only as required by PLOS GPH. The direction of the arrows indicate causality between variables and is described in the figure legend. |
| 40 | Confuse image | This figure is the Hypothesised causal pathway of how perceived severity of leptospirosis leads to *Leptospira* seropositivity in men and women, and provides a visual description of analysis 1 and 2. The supporting figure legend that describes this image can be found in the main text from line 263, describing the figure. |
| 41 | Individuals? | Figure 4 legend updated on line 356 to define 'HH' and 'n' to clarify what is meant by households and individuals |
| 41 | Insert percentages | Added and updated figure legend 4 on line 357 - "Percentages are calculated relative to the total of the nearest previous level" |
| 41 | Insert Fig 4 main informations on the abstract | Added number of men and women into abstract on line 59. |
| 42 | Insert the average of all ages together | An additional plot has been added to figure 5 showing average seroprevalence across all ages. |
| 42 | Was there comparisons between males and females? | Across the manuscript, comparisons are made within female or male groups to identify exposure pathways relevant to each group. Differences between men and women can be found descriptively in Table 1, and we tested for differences between groups using effect modification tests in Table 2. For example, we found that sex was an effect modifier for the relationship between perceived severity and walking barefoot. |
| 43 | Really? What can explain women walking barefoot more than men? | This odds ratio is not derived from women vs men, but women who perceived leptospirosis as extremely serious vs those that perceived leptospirosis as less serious. This is explained in figure legend 6 on line 460. |
| 43 | Are there differences between men of different age ranges? That can help direct health education interventions | Thank you for this insightful suggestion. We conducted a sub analysis based on this suggestion and found that the association of perceived severity with walking barefoot and walking through sewage water differed across ages in men. Specifically, we observed that the relationship between perceived disease severity and high-risk behaviours varied across age groups in males. Men aged 31–45 years who perceived leptospirosis as extremely serious had lower odds of engaging in risky behaviours, such as walking barefoot or through sewage water. Similarly, men aged 18–30 years who perceived leptospirosis as extremely serious also showed lower odds of walking barefoot.  The sub-analysis investigating the associations of perceptions with high risk behaviours across different age ranges in men and women has been added to the supplementary information 4a-c, and has been described in the main text in the methods section (line 319), results (line 376 and 484) and discussion (line 554). |
